# Inhibiton of tumor-derived prostaglandin-e2 prevents the induction of human myeloid-derived suppressor cells (MDSCs) and rescues anti-tumor immunity

**DOI:** 10.1186/2051-1426-2-S3-P224

**Published:** 2014-11-06

**Authors:** Yumeng Mao, Dhifaf Sarhan, Isabel Poschke, Andreas Lundqvist, Rolf Kiessling

**Affiliations:** 1 Karolinska Institutet, Stockholm, Sweden; 2 German Cancer Research Center (DKFZ), Heidelberg, Germany

## Background

Cancer progression is associated with a severe impairment of anti-tumor immune responses. The accumulation of peripheral blood CD14+HLA-DRlo/- monocytic MDSCs has been identified as a prognostic factor in advanced stage melanoma patients. These cells suppress T cell functions *ex vivo *and reversely correlate with the frequencies of antigen-specific T cells in patients. However, the precise mechanistic basis underlying this effect is unclear, particularly with regard to how MDSCs are induced and how they could limit functions of NK cells in cancer patients.

## Methods

The effects of tumor-derived factors in inducing phenotypic and functional alternations on healthy human monocytes or CD34+ hematopoietic stem cells (HSCs) were analyzed in vitro, by co-culturing monocytes with early-passage tumor cells or addition of tumor-conditioned medium, respectively. Suppression of T or NK cell activity by MDSC-like cells was compared with that of freshly isolated CD14+HLA-DRlow/- monocytic MDSCs (moMDSCs) from patients with melanoma. In addition, to explore the in vivo relevance of targeting PGE2 to reduce MDSC expansion, we established a murine model, where tumor cells were disabled from cyclooxygenase-2 (COX-2) production and in vivo NK cytotoxicity was evaluated by live imaging.

## Results

We recently demonstrated that tumor-derived factors promoted monocytes to acquire an MDSC-like phenotype and suppressive functions of T cells via COX-2/PGE2 pathway (Mao et al. Cancer Res., 2013). Moreover, tumor-derived factors resulted in a retention of c-kit+ cells (p < 0.05) and hampered differentiation of CD34+ HSCs to functional myeloid cells. As a result, these cells showed decreased ability to stimulate T cells to proliferate and produce IFNγ (p < 0.05). In patients with melanoma, freshly isolated CD14+HLA-DRlow/- moMDSCs strongly suppressed cytotoxicity (p < 0.05) and production of IFNg (p < 0.01) by NK cells, through production of TGFβ (p < 0.05). In vitro, binding of PGE2 to EP2 or EP4 receptors on monocytes activated the p38MAPK/ERK pathway and resulting in elevated secretion of TGFβ (p < 0.05) and reduced NK cell cytotoxicity (p < 0.01). Importantly, silencing COX-2 in 4T1 tumor cells reduced the frequency of splenic CD11b+Gr1+ MDSCs (p < 0.01), resulting in concomitant improved in vivo clearance of NK-sensitive YAC-1 target cells (p < 0.05).

## Conclusions

Collectively, our results reveal a direct involvement and molecular machinery of tumor-derived factors, in particular PGE2, in inducing immature phenotype and stimulating suppressive functions from MDSCs. Therefore, combining COX-2 inhibitors with immunotherapy may result to a favorable clinical outcome in patients with cancer.

